# Editorial: Highlights in diabetes and pregnancy

**DOI:** 10.3389/fcdhc.2025.1736188

**Published:** 2025-11-21

**Authors:** A. Seval Ozgu-Erdinc

**Affiliations:** Perinatology Department, University of Health Sciences Bilkent City Hospital, Ankara, Türkiye

**Keywords:** gestational diabetes mellitus, thyroid disease, neonatology, KAP, physical activity, pregnancy outcome, saliva, feeding

Diabetes during pregnancy constitutes a pivotal intersection of maternal and fetal health, presenting both acute obstetric challenges and enduring metabolic repercussions that transcend generations. With the global prevalence of diabetes rising alongside obesity and metabolic syndrome, its influence on reproductive health has assumed unprecedented significance. This Research Topic, “Advances in Diabetes and Pregnancy,” brings together state-of-the-art research that deepens our understanding of the underlying mechanisms, diagnostic innovations, therapeutic strategies, and long-term implications of diabetes manifesting before or during gestation.

Once regarded as a transient gestational complication, gestational diabetes mellitus (GDM) is now increasingly acknowledged as a sentinel event reflecting lifelong endocrine and metabolic vulnerability. The studies presented here challenge the conventional perspective, providing robust evidence that diabetes in pregnancy—whether gestational or pregestational—serves as a critical lens through which systemic metabolic risk and developmental programming can be observed. Through comprehensive epidemiologic analyses, interventional studies, and biomarker-based research, this Research Topic illuminates the multifaceted dimensions of diabetes in pregnancy and calls for a paradigm shift toward continuous, multidisciplinary management.

Perhaps the most profound ramifications of maternal diabetes manifest in the offspring through mechanisms of fetal programming and epigenetic modification. The Developmental Origins of Health and Disease (DOHaD) paradigm posits that maternal hyperglycemia during gestation can induce lasting alterations in fetal physiology and metabolism. Azuma et al. explored these effects through an innovative salivary transcriptomic approach, analyzing gene expression of appetite-regulating markers—PRKAA2, GHRL, POMC, and NPY2R—in neonates born to diabetic mothers. The study revealed a distinctive expression profile: downregulation of ghrelin and PRKAA2 coupled with upregulation of POMC, correlating with greater neonatal adiposity and feeding difficulties. These molecular changes imply that maternal hyperglycemia may reprogram hypothalamic circuits controlling appetite and energy balance, predisposing infants to future metabolic disorders. Salivary transcriptomics thus emerges as a promising non-invasive biomarker platform for early identification of infants at risk for metabolic dysregulation. Early detection could facilitate targeted nutritional and behavioral interventions aimed at mitigating long-term health consequences. Longitudinal studies following infants of diabetic mothers into later life are essential to fully elucidate these transgenerational effects.

Cui et al. conducted a systematic review and meta-analysis of 17 studies (15 randomized controlled trials and 2 cohort studies), demonstrating that structured antenatal exercise significantly reduced rates of macrosomia, preterm birth, cesarean delivery, fetal growth restriction, and birth trauma in women with GDM. Interestingly, single-component interventions—such as aerobic exercise alone—yielded superior neonatal outcomes compared with combined regimens, possibly reflecting greater adherence and more targeted physiological effects. Exercise exerts multifactorial benefits in GDM by improving insulin sensitivity, enhancing cardiovascular dynamics, modulating placental nutrient transfer, and reducing systemic inflammation. Despite this evidence, real-world implementation remains inconsistent. These findings emphasize the urgent need for standardized, evidence-based exercise prescriptions tailored to individual risk profiles and supported by multidisciplinary teams. Integrating structured physical activity programs into prenatal care represents a cost-effective and scalable intervention to optimize perinatal outcomes in GDM.

The long-term consequences of GDM for maternal health extend far beyond the risk of subsequent type 2 diabetes. In a landmark population-based study, Shih et al. utilized the TriNetX U.S. Collaborative Network, encompassing over 300,000 pregnancies with up to two decades of follow-up. Through rigorous propensity score matching, the authors demonstrated that women with prior GDM exhibited significantly elevated risks of developing diverse thyroid disorders—including hyperthyroidism, hypothyroidism, thyroiditis (notably Hashimoto’s), and both toxic and non-toxic goiter. Remarkably, these risks persisted up to 20 years postpartum, underscoring that GDM signifies systemic endocrine vulnerability rather than an isolated metabolic derangement. These insights advocate for incorporating long-term thyroid monitoring into post-gestational care, particularly among older or obese women. Redefining GDM as a multisystemic endocrine disorder rather than a transient glucose imbalance demands a comprehensive approach encompassing extended endocrinologic evaluation and lifelong surveillance. The thyroid–GDM association likely stems from shared pathophysiological underpinnings such as chronic inflammation, autoimmune mechanisms, or genetic predisposition—areas warranting further research. While the epidemiologic burden of GDM is well recognized, compelling evidence highlights the potential for modifiable interventions to mitigate adverse outcomes.

The study of Yang et al. assesses the knowledge, attitudes, and practices (KAP) of neonatologists in Hubei Province, China, concerning long-term complications in infants born to mothers with gestational diabetes mellitus (GDM). Utilizing a robust, validated 28-item survey, over 1,600 board-certified neonatologists with clinical experience managing GDM-exposed newborns participated. Results reveal that while a vast majority demonstrate high knowledge levels (89%) and positive attitudes (94%) towards managing GDM-related neonatal risks, their adherence to recommended clinical practices is comparatively lower (72%). Gaps were noted particularly in consistent follow-up evaluations and specialist referrals. Factors such as age, clinical experience, and practice setting influenced competency and practice patterns. The study highlights the need for targeted educational programs, enhanced guideline dissemination, and system-level interventions to bridge the knowledge-practice gap. These efforts could improve early identification and management of metabolic and developmental risks in GDM-exposed infants, ultimately reducing long-term adverse health outcomes. This research contributes valuable insights into neonatology care for GDM and provides evidence to inform policy and clinical improvements in maternal-child health.

## Conclusion

This Research Topic reinforces the need to reconceptualize GDM as a sentinel metabolic condition with lifelong implications for both mother and child. The complexity of diabetes in pregnancy encompasses intertwined biological pathways—ranging from placental endocrine signaling and metabolic crosstalk to epigenetic reprogramming—that demand integrative investigation.

Despite notable progress, unresolved questions persist regarding optimal management thresholds, long-term intervention efficacy, and the biological underpinnings of transgenerational risk. Addressing these gaps will require sustained interdisciplinary collaboration among endocrinologists, obstetricians, neonatologists, molecular scientists, and public health experts.

By embedding endocrinologic screening, structured exercise programs, novel biomarker surveillance, and comprehensive postpartum care into routine practice, the field can move toward a holistic, evidence-driven model of maternal-fetal medicine. The studies featured in this Research Topic collectively advance this goal, offering valuable insights that will inform clinical practice and stimulate future innovation in the management of diabetes in pregnancy.

